# Inguinal lymph node metastases from a testicular seminoma: a case report and a review of the literature

**DOI:** 10.1186/1752-1947-4-378

**Published:** 2010-11-25

**Authors:** Mohamed Ismail, Faruquz Zaman, Sohail Baithun, Venod Nargund, Jhumur Pati, Junaid Masood

**Affiliations:** 1 Department of Urology, Homerton University Hospital NHS Foundation Trust, London, E9 6SR, UK; 2 Department of Pathology, Bart's and the London NHS Trust, London, EC1A 7BE, UK; 3 Department of Urology, Bart's and The London NHS Trust, London EC1A 7BE, UK

## Abstract

**Introduction:**

We report the case of a true hermaphrodite with testicular seminoma with resulting metastases to the inguinal lymph nodes eight months after radical orchidectomy. This is an unusual presentation of testicular cancer and, to the best of our knowledge, the first report of this kind in the literature.

**Case presentation:**

A 45-year-old Caucasian true hermaphrodite, raised as a male, developed a testicular seminoma. He had undergone a left orchidopexy at the age of 10 for undescended testes. Metastases from testicular tumors to inguinal lymph nodes are a rare occurrence. It has been suggested that previous inguinal or scrotal surgery may alter the pattern of nodal metastasis of testicular cancer. We review the literature to evaluate the incidence of inguinal lymph node involvement in early stage testicular cancer and discuss possible routes of metastases to this unusual site. We also discuss the management of the inguinal lymph nodes in patients with testicular tumors and a previous history of inguinal or scrotal surgery, as this remains controversial.

**Conclusion:**

Inguinal lymph node metastases from testicular cancer are rare. A history of inguinal or scrotal surgery may predispose involvement of the inguinal nodes. During radical inguinal orchidectomy, the surgeon should be careful to minimize the handling of the testis and ensure high ligation of the spermatic cord up to the internal inguinal ring to reduce the risk of inguinal lymph node metastasis.

## Introduction

Testicular cancer is a relatively rare cancer and is responsible for one to two percent of all male cancer. In the UK, around 2000 new cases are diagnosed every year [1]. Seminoma is the most common of the germ cell tumors (GCTs) that affect the testis. It constitutes around 40 to 45 percent of all GCTs. Histologically it can be subdivided into classic, anaplastic and spermatocytic subtypes [2]. Testicular seminoma has rarely been reported in patients with true hermaphroditism [3].

Usually, the testicular lymphatics drain along the gonadal vessels to the retroperitoneal nodes, which are located between the lower thoracic and lumbar vertebrae, including the renal hili and around the inferior vena cava and the aorta [4]. The lymphatics that accompany the testicular vessels exit the testis through the inguinal ring to the retroperitoneal para-aortic lymph nodes following typical patterns of spread according to the side of the primary tumor [5]. Involvement of the iliac and inguinal nodes can occasionally occur in a secondary retrograde fashion, usually when there are bulky retroperitoneal metastases [4].

Primary involvement of the iliac and inguinal nodes is rare and associated with tumor extension into the epididymis, breaching of the tunica vaginalis through to the scrotal wall or extension to the vas deferens. Direct inguinal metastases are also reported as a result of previous surgical manipulation of the inguinoscrotal region [6], as in our case.

Usually the superficial inguinal nodes drain the skin from the lower abdomen, part of the buttocks and scrotum, the perineum and the penis. The deep inguinal nodes, which can be found under the fascia lata, are drained from the superficial nodes, legs and deep penile structures. However, following surgery where the testicular lymphatics are damaged and disrupted as a result of dissection of the spermatic cord during orchidopexy, orchidectomy, hydrocele repair, varicocelectomy or hernia repair, these lymphatics seek new collateral vessels for drainage. Injured lymphatics from scrotal incisions re-anastomose with the testicular lymphatics and can therefore provide a direct route of spread to the inguinal nodes [7].

Ohtani and Gannon studied the microvasculature of the rat vas deferens and have described the arterial and venous drainage in great detail [8]. They found a subepithelial capillary network and it has been postulated that this capillary network exists in humans. Lockett *et al*. postulated in his report that seminoma may have spread along a similar subepithelial capillary network along the vas [9]. For these reasons, radical inguinal orchidectomy is the procedure of choice for testicular tumors to avoid the sequelae associated with scrotal contamination.

## Case presentation

**Figure 1:**
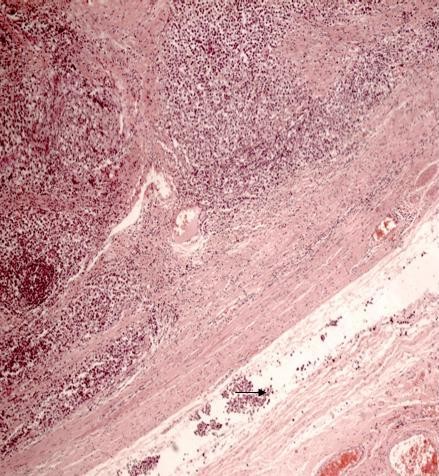
**A histology specimen shows classical seminoma arising in the testis**. Vascular and perineural invasion can be seen (arrow). The spermatic cord margin was free of tumor.

**Figure 2:**
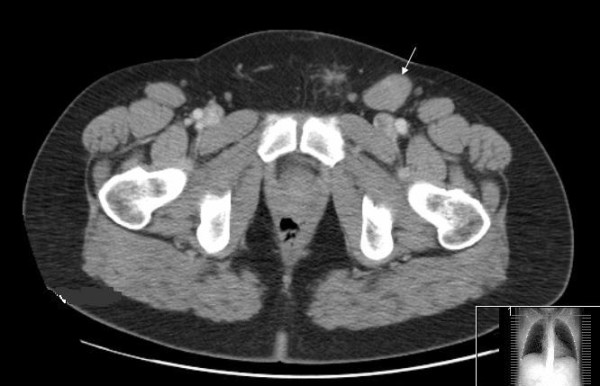
**A CT scan of the pelvis revealing a 2.4 cm left inguinal lymph node (arrow)**.

**Figure 3:**
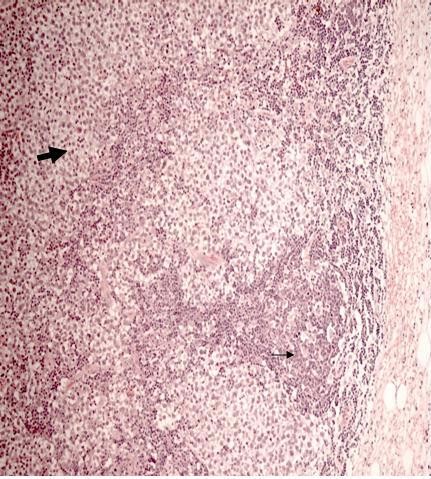
**Metastases to the inguinal node consistent with the original seminoma (large arrow) and invasion through the capsule into the surrounding adipose tissue (small arrow)**.

A 45-year-old Caucasian true hermaphrodite, who has been raised as a male, presented with a hard left testicular mass which had significantly increased in size over the preceding few months. His past medical history included a left orchidopexy at the age of 10 years. He had also previously undergone a hysterectomy and a right oophorectomy and no testicular tissue had ever been identified on the right side. On examination, he had a left-sided inguinoscrotal scar. His left testis was enlarged and hard. We could detect no other abnormality. His human chorionic gonadotrophin (HCG) level was elevated (11 mIU/ml) and the other tumor markers, including lactate dehydrogenase (LDH - 240 U/L) and alpha-fetoprotein (AFP - 3 ng/ml), were normal. A staging computed tomography (CT) scan showed no evidence of metastatic disease. A left radical orchidectomy was performed. Intra-operatively, his whole testis was found to be hard and no distinct mass was identified. A histopathology examination revealed a homogenous friable testis with no epididymis identified. The tumor breached the tunica albuginea and tunica vaginalis. Microscopic examination showed a classical seminoma with vascular and perineural invasion (Figure 1). The spermatic cord margin appeared free of the tumor and the tumor reached the excision margin. Therefore, histological staging demonstrated a T2 lesion. His HCG level was normal after the orchidectomy. He was commenced on a testosterone replacement therapy post-operatively. At a routine eight month follow up, he was found to have an enlarged lymph node in the left inguinal region. A CT scan confirmed the presence of a 2.4 cm left inguinal lymph node (Figure 2). There was also pelvic lymph node and chest involvement. An excision biopsy of his inguinal node revealed a classical metastatic seminoma with extra-capsular spread to the surrounding adipose tissue (Figure 3). Treatment was started with two cycles of carboplatin AUC10. He made a good recovery after the chemotherapy and repeat CT scans have shown no evidence of recurrence after two years of follow up.

## Discussion

In patients with a prior history of orchidopexy or scrotal surgery who have a testicular tumor, the incidence of inguinal metastases is unclear but has been reported in series varying from two percent [10] up to 10 percent [11]. Daugaard *et al*. evaluated the incidence of inguinal lymph node metastases in 695 patients with stage I testicular cancer [10]. Two percent of patients developed inguinal node metastasis. Non-seminomatous GCTs more frequently invaded inguinal lymph nodes than seminoma.

The routine management of the inguinal lymphatics (palpable or not) in patients with testicular tumors and a previous history of inguinal or scrotal surgery remains controversial, as a result of insufficient data [6]. Prophylactic inguinal lymphadenectomy is rarely mentioned in the literature. In some series, patients have been found to have positive inguinal nodes with no retroperitoneal lymphadenopathy, supporting the need to perform routine ipsilateral inguinal lymphadenectomy even when the retroperitoneal nodes are clear [6,12]. Wheeler *et al. *advocated ipsilateral inguinal and bilateral retroperitoneal node dissection as the primary therapy for non-seminomatous testicular tumor with a previous history of scrotal and inguinal procedures [6].

Another series in which 20 cases of testicular tumor and previous scrotal surgery were presented, failed to document the incidence of inguinal lymphadenopathy [13]. They concluded that additional treatment to the inguinal nodes was not required but most of their patients underwent immediate radiation therapy or chemotherapy with none undergoing groin dissection. The true incidence of inguinal metastases in their study is therefore unknown. It was suggested that failure to perform prophylactic inguinal node dissection does not adversely affect patient survival and regular groin palpation and dissection of any suspicious lymph nodes was recommended. If positive, cisplatinum, vinblastine and bleomycin chemotherapy is given [14]. Mianne *et al*. also suggested that prophylactic ipsilateral inguinal dissection is not necessary in patients with non-seminomatous testicular tumors with a history of inguinal or scrotal surgery, owing to the efficacy of primary and secondary chemotherapy [15]. However, for testicular seminoma they advocated additional inguinoscrotal radiotherapy. The low incidence of inguinal lymph node metastasis, morbidity rate following radical ilioinguinal dissection, the accessibility of the inguinal nodes to follow-up examination and the availability of highly successful multimodal therapy make expectant management of the clinically negative groin an attractive alternative. A diagnosis of inguinal node metastases is usually made by an excision biopsy of the nodes, but fine needle aspiration (FNA) has also been used.

## Conclusion

Inguinal lymph node metastases from testicular cancer are rare. A history of inguinal or scrotal surgery may predispose involvement of the inguinal nodes as a result of altered patterns of lymphatic drainage. The routine management of inguinal lymphatics (palpable or not) in patients with testicular tumors and a previous history of inguinal or scrotal surgery remains controversial, with no consensus amongst those treating these patients. During radical inguinal orchidectomy, the surgeon should be careful to minimize the handling of the testis and ensure high ligation of the spermatic cord up to the internal inguinal ring to reduce the risk of inguinal lymph node metastasis.

## Abbreviations

(AFP): Alpha-fetoprotein; (CT): Computed tomography; (FNA): Fine needle aspiration; (GCTs): Germ cell tumors; (HCG): Human chorionic gonadotrophin; (LDH): Lactate dehydrogenase

## Consent

Written informed consent was obtained from the patient for publication of this case report and any accompanying images. A copy of the written consent is available for review by the Editor-in-Chief of this journal.

## Competing interests

The authors declare that they have no competing interests.

## Authors' contributions

MI wrote the original manuscript. FZ and JP analyzed and interpreted the patient data with regard to the hematological and radiological diagnosis. SB performed the histological examination of the testis. VN and JM were major contributors in writing the manuscript. All authors read and approved the final manuscript.
